# Impact of nanopore-based metagenome sequencing on tick-borne virus detection

**DOI:** 10.3389/fmicb.2023.1177651

**Published:** 2023-05-30

**Authors:** Koray Ergunay, Ender Dincer, Silvia A. Justi, Brian P. Bourke, Suppaluck P. Nelson, Hsiao-Mei Liao, Mehmet Ozkan Timurkan, Bekir Oguz, Ismail Sahindokuyucu, Omer Faruk Gokcecik, Drew D. Reinbold-Wasson, Le Jiang, Nicole L. Achee, John P. Grieco, Yvonne-Marie Linton

**Affiliations:** ^1^Walter Reed Biosystematics Unit (WRBU), Smithsonian Institution, Museum Support Center, Suitland, MD, United States; ^2^One Health Branch, Walter Reed Army Institute of Research (WRAIR), Silver Spring, MD, United States; ^3^Department of Entomology, Smithsonian Institution–National Museum of Natural History (NMNH), Washington, DC, United States; ^4^Department of Medical Microbiology, Virology Unit, Faculty of Medicine, Hacettepe University, Ankara, Türkiye; ^5^Department of Virology, Faculty of Veterinary Medicine, Dokuz Eylül University, Izmir, Türkiye; ^6^Naval Medical Research Center (NMRC), Silver Spring, MD, United States; ^7^Henry M. Jackson Foundation for the Advancement of Military Medicine, Bethesda, MD, United States; ^8^Department of Virology, Faculty of Veterinary Medicine, Ataturk University, Yakutiye, Erzurum, Türkiye; ^9^Department of Parasitology, Faculty of Veterinary Medicine, Van Yuzuncu Yil University, Van, Türkiye; ^10^Bornova Veterinary Control Institute, Veterinary Control Institute Directorates, Ministry of Agriculture and Forestry, Izmir, Türkiye; ^11^U.S. Army Medical Research Diriectorate-Georgia (USAMRD-G), Tbilisi, Georgia; ^12^Department of Biological Sciences, Eck Institute for Global Health, University of Notre Dame, Notre Dame, IN, United States

**Keywords:** nanopore, tick, tick-borne, metagenome, virus, zoonoses

## Abstract

**Introduction:**

We evaluated metagenomic nanopore sequencing (NS) in field-collected ticks and compared findings from amplification-based assays.

**Methods:**

Forty tick pools collected in Anatolia, Turkey and screened by broad-range or nested polymerase chain reaction (PCR) for Crimean-Congo Hemorrhagic Fever Virus (CCHFV) and Jingmen tick virus (JMTV) were subjected to NS using a standard, cDNA-based metagenome approach.

**Results:**

Eleven viruses from seven genera/species were identified. Miviruses Bole tick virus 3 and Xinjiang mivirus 1 were detected in 82.5 and 2.5% of the pools, respectively. Tick phleboviruses were present in 60% of the pools, with four distinct viral variants. JMTV was identified in 60% of the pools, where only 22.5% were PCR-positive. CCHFV sequences characterized as Aigai virus were detected in 50%, where only 15% were detected by PCR. NS produced a statistically significant increase in detection of these viruses. No correlation of total virus, specific virus, or targeted segment read counts was observed between PCR-positive and PCR-negative samples. NS further enabled the initial description of Quaranjavirus sequences in ticks, where human and avian pathogenicity of particular isolates had been previously documented.

**Discussion:**

NS was observed to surpass broad-range and nested amplification in detection and to generate sufficient genome-wide data for investigating virus diversity. It can be employed for monitoring pathogens in tick vectors or human/animal clinical samples in hot-spot regions for examining zoonotic spillover.

## 1. Introduction

Genomic identification of microbial pathogens has established a substantial role in the diagnosis and monitoring of infectious diseases within the One Health concept (Trinh et al., 2018). Metagenomic investigations, facilitated by the widespread use of next-generation sequencing (NGS), enable the analysis of the nucleic acid content of any sample, without prior information on pathogens. In clinical diagnosis, metagenomic testing has proven useful in infections with unconventional agents, non-specific clinical presentation, and in instances where pathogen diversity hampers targeted detection (Dulanto Chiang and Dekker, 2020). Metagenome sequencing can further be employed for environmental surveillance to identify pathogen spillover. As the majority of emerging infections originate from wildlife and adapt to domestic animals to infect humans via spillover events, identification of potential zoonotic pathogens in the animal–human interface may facilitate the description of agents with imminent public health impact (Quer et al., 2022). Another use of metagenome sequencing in emerging diseases involves bio- or xeno-surveillance where blood-sucking arthropods, such as mosquitoes, ticks, and sandflies, can be used as sentinels to screen pathogens encompassing multiple hosts (Brinkmann et al., 2016).

Sequencers based on third-generation technology including nanopore sequencing (NS) are among the most widely used NGS platforms, due to their relatively low cost and portability (Kumar et al., 2017). Based on single-molecule synthesis, NS produces longer reads and allows real-time data access, significantly reducing the time required for sequencing (Petersen et al., 2019). Despite limitations of depth and accuracy, NS holds potential as a point-of-care or field-friendly metagenomics platform due to its flexibility (Greninger et al., 2015; Quick et al., 2016; Russell et al., 2018). NS can be particularly useful in monitoring arthropod-borne viruses in vectors or reservoirs, especially in regions with probable epizootic events. Viruses involved in spillover events often carry RNA genomes and exhibit high mutation rates, requiring broad-range or high-fidelity primer sets for detection by standard amplification techniques, advocating further for an inclusive approach provided by metagenomics for surveillance. NS has been employed to characterize virus genomes in mosquito pools screened by targeted amplification (Russell et al., 2018) and has proven capable of detecting mosquito-borne viruses in a single infected sample under controlled conditions (Batovska et al., 2017). Nevertheless, it has been rarely used to identify tick-borne viruses in a clinical or surveillance setting. This study aimed to evaluate NS-based metagenome sequencing in ticks, screened by polymerase chain reaction (PCR).

## 2. Materials and methods

### 2.1. Samples

The study cohort comprised 40 tick pools, collected in Anatolia, Turkey during 2020–2021 (Supplementary Table 1) (Dinçer et al., 2022). Individual adult ticks had been collected from infested animals including cattle (*Bos taurus*), sheep (*Ovis aries*), and dogs (*Canis familiaris*). They were morphologically identified to species level using appropriate taxonomic keys (Filippova, 1997; Walker et al., 2000, 2003; Estrada-Pena et al., 2004; Apanaskevich and Horak, 2008), pooled into groups of 4–12 individuals according to the collection site and species, and then stored at −80°C. The pools were macerated by vortexing with beads in Eagle's minimum essential medium supplemented with 5% fetal bovine serum, centrifuged at 4,000 rpm for 4 min. The supernatants from the pools were subsequently collected and subjected to nucleic acid purification by High Pure Viral Nucleic Acid Kit (Roche Diagnostics, Mannheim, Germany) and complementary DNA (cDNA) synthesis with random hexamers, using the RevertAid First-Strand cDNA Synthesis Kit (Thermo Fisher Scientific, Hennigsdorf, Germany), as directed by manufacturer's protocol. The pools were screened for generic nairovirus and Jingmen tick virus (JMTV) by previously described in-house PCR assays (Honig et al., 2004; Yu et al., 2020), utilizing identical conditions. The generic nairovirus PCR targeted the central motif A of the viral replicase, encoded by the genome segment L (Honig et al., 2004), whereas viral NS5-like protein on segment 1 was targeted by the JMTV assay in a nested format (Yu et al., 2020).

### 2.2. Nanopore sequencing (NS)

A fresh aliquot from the processed tick pool was used for sequencing. Briefly, the aliquot was lysed in ATL-DX lysis buffer with Precellys zirconium oxide beads (Bertin Corp., Rockville, MD, USA) using Bullet Blender 24 Gold (Next Advance, Troy, NY, USA). The lysate was centrifuged, and the supernatant was extracted using the IndiMag Pathogen Kit (Indical Bioscience, Leipzig, Germany) with KingFisher™ Flex Purification System, (Thermo Fisher Scientific, Waltham, MA, USA) according to the manufacturer's protocol. Purified nucleic acids were treated with ezDNase (TFS) and subjected to cDNA synthesis using NEBNext Ultra II RNA First-Strand and Non-Directional RNA Second Strand Synthesis modules, utilizing random primer mix (New England Biolabs, Ipswich, MA, USA) according to manufacturer recommendations. Double-stranded cDNA was cleaned up using Agencourt AMPure XP reagent (Beckman Coulter Biosciences, Indianapolis, IN, USA) and quantified using the Qubit dsDNA HS Assay Kit (TFS).

A Ligation Sequencing Kit (Oxford Nanopore Technologies, Oxford, UK) and NEBNext End repair and Quick Ligation Modules (NEB) were used as directed by the manufacturer's protocol. Libraries were quantitated by Qubit (TFS). Samples were barcoded with the Rapid Barcoding Kit 96 (ONT) for combined sequencing. An epMotion 5075 (Eppendorf, Hamburg, Germany) was used for automated liquid handling. Sequencing libraries, combined as 16 and 24 barcoded pools, were loaded on GridION Mk1C (ONT) sequencer and run for 72 h.

Basecalling and demultiplexing were performed on the GridION with the MinKNOW operating software v21.11.7 (ONT) and Guppy v 5.1.13 (Wick et al., 2019). Raw reads were trimmed with Porechop to remove adapter sequences and then filtered with NanoFilt to remove reads with q-scores ≤ 9 and read lengths ≤ 100 bp (Wick et al., 2017; De Coster et al., 2018). This data was then cleaned by removing any tick host DNA using Minimap2 v2.24 and Samtools v1.9 (Li, 2018; Danecek et al., 2021).

### 2.3. Sequence data and statistical analysis

The processed reads were aligned to the National Center for Biotechnology Information (NCBI) Reference Sequence (RefSeq) database using DIAMOND v2.0.14 (Buchfink et al., 2021), visualized using MEGAN6 (v6.23.2) (Huson et al., 2016). Taxon-based read counts were obtained from MEGAN6. Sequences were handled using Geneious Prime (v2022.2.1) (Biomatters Ltd., Auckland, New Zealand). The BLASTn algorithm was used for similarity searches in the NCBI database (Altschul et al., 1990). Virus read mapping was carried out using Minimap2 plug-in for Geneious Prime, with default settings optimized for nanopore data, using BLASTn hits with highest identity scores as a reference. Optimal substitution models on individual alignments were estimated by MEGAX, which was further employed to infer evolutionary history according to the Bayesian information criterion (Kumar et al., 2018).

*T*-test and Wilcoxon signed rank test were used based on the F-test results to assess the variance of the groups being compared. For this purpose, the samples were separated into PCR-negative and PCR-positive groups for each virus. Within these groups, the total number of reads generated per sample by NS, total virus reads, specific virus reads (JMTV or CCHFV), and target segment reads (for each PCR) were individually recorded, and proportions relative to the total number of reads or specific virus reads were calculated. Furthermore, the samples were compared in a binomial manner, where the results were scored as either 0 (PCR/NS negative) or 1 (PCR/NS positive). The Z-statistics obtained were then compared to the critical values for 95 and 99% two-tailed tests (at 1.96 and 2.58, respectively).

## 3. Results

### 3.1. Virus detection

A total of 301 ticks were sequenced in 40 pools, which comprised *Rhipicephalus bursa* (70%), *R. sanguineus* sensu latu (17.5%), *R. turanicus* (7.5%), and *Haemaphysalis parva* (2.5%) species (Table 1). The sequencing runs produced total and virus reads in the range of 265–343,857 (mean: 37,853.2, SD: 76,767.1) and 3–19,148 (mean: 1,071.7, SD: 3,236.3), respectively (Supplementary Table 1). A total of 11 viruses from seven genera or species were identified (Table 1). Sequences from multiple viruses were observed in 31 pools (77.5%). In samples with a single detectable virus (nine pools), Bole tick virus 3 (BTV3) (genus *Mivirus*, species *Mivirus boleense*) comprised the majority of the mapped reads (7/9, 77.8%).

**Table 1:** Virus detection by NS in pooled ticks.

**Viruses**		**Tick pools**				
		***R. bursa (n: 28, 70%)***	***R. sanguineus (n: 7, 17.5%)***	***R. turanicus (n: 3, 7.5%)***	***Hae. parva (n: 2, 5%)***	***Total***
*Mivirus*	Bole tick virus 3	28	3	1	1	33 (82.5%)
	Xinjiang mivirus 1	0	1	0	0	1 (2.5%)
*Mogiani tick virus*	Jingmen tick virus	17	3	2	2	24 (60%)
*Phlebovirus*	Brown dog tick phlebovirus 2	12	4	1	0	17 (42.5%)
	Phlebovirus Strandja	3	0	0	0	3 (7.5%)
	Phlebovirus Anatolia	0	2	1	0	3 (7.5%)
	Lesvos virus	0	0	0	1	1 (2.5%)
*Nairovirus*	Crimean-Congo hemorrhagic fever virus	12	6	0	2	20 (50%)
*Peribunyavirus*	*Ixodes ricinus* bunyavirus-like virus 1	1	0	0	0	1 (2.5%)
*Orthomyxovirus*	Quaranjavirus	0	1	0	0	1 (2.5%)
Unclassified *Riboviria*	Butler's Creek virus	0	0	0	1	1 (2.5%)

### 3.2. JMTV and CCHFV findings

**Figure 1:**
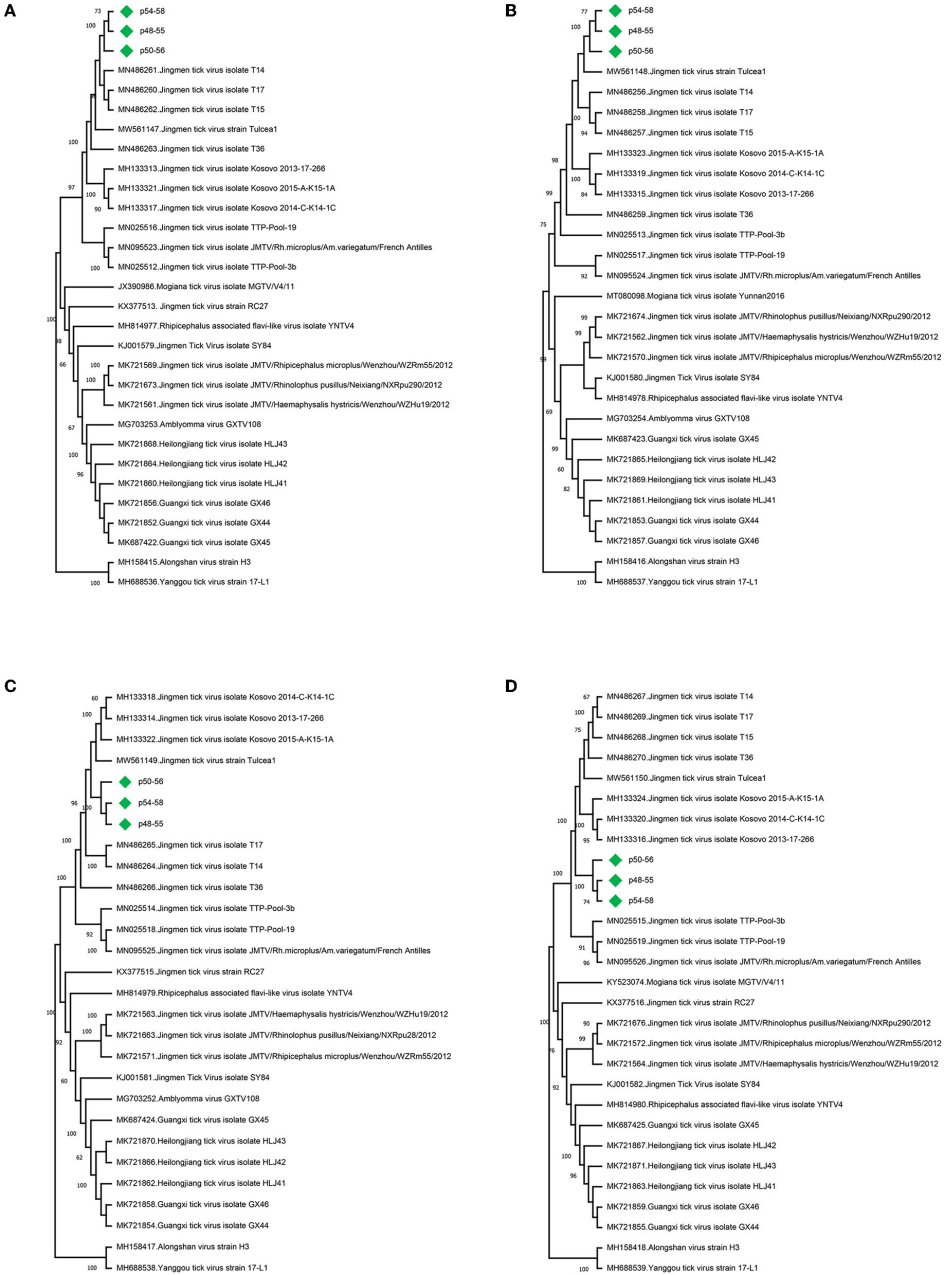
Maximum likelihood analysis of the Jingmen tick virus (JMTV) genome sequences [**(A)** segment 1,1084 bp; **(B)** segment 2, 2,325 bp; **(C)** segment 3, 1,247 bp, **(D)** segment 4, 2,293 bp]. The tree is constructed for 500 replications using the Tamura–Nei, gamma distributed with invariant sites (segments 1 and 3) or the gamma distributed, general time reversible (segments 2 and 4) models. Viruses are indicated by GenBank accession number, name, and isolate/strain identifier. The sequences obtained in the study are marked. Bootstrap values higher than 60 are provided.

Jingmen tick virus (family *Flaviviridae*, species *Mogiana tick virus*) sequences were identified in 24 pools (60%) (Table 1) (Simmonds et al., 2019). JMTV is a segmented RNA virus documented as a causative agent of febrile disease associated with tick bites in humans (Jia et al., 2019), sometimes co-detected with CCHFV in severe cases (Emmerich et al., 2018). JMTV and related viruses are widely distributed in Eurasia and the Americas with virus RNA in bats, cattle, primates, and rodents, in addition to ticks and mosquitoes (Temmam et al., 2019; Guo et al., 2020). In the tick pools, we observed mapped read or contig counts of 1–10,348, with 90.2–99.4% identities to previously reported JMTV sequences (Supplementary Table 2). Near-complete open reading frame (ORF) contigs from all four genome segments were available in certain pools, where phylogenetic analysis of genome segments revealed clustering and shared ancestors with JMTVs reported from Turkey and the Balkans (Figure 1).

**Figure 2:**
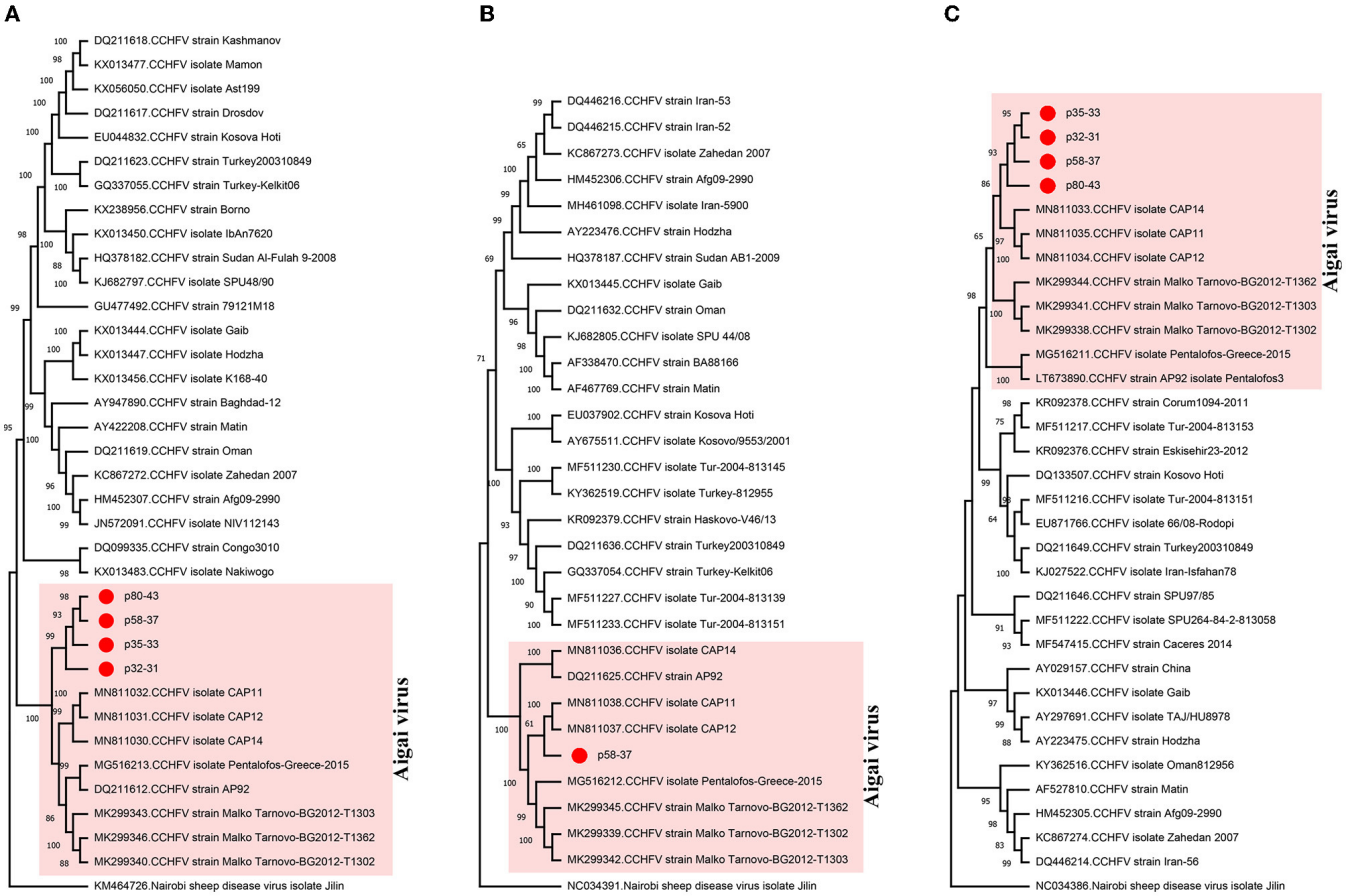
Maximum likelihood analysis of the Crimean–Congo hemorrhagic fever virus (CCHFV) genome sequences [**(A)** L segment, 12,887 bp; **(B)** M segment, 3,765 bp; **(C)** S segment, 1,361 bp]. The tree is constructed for 500 replications using the general time reversible, gamma distributed with (M segment) or without invariant sites (L segment) or Kimura 2-parameter models (S segment). Viruses are indicated by GenBank accession number, abbreviation, and isolate/strain identifier. The sequences obtained in the study are marked. Bootstrap values higher than 60 are provided. Nairobi sheep disease virus serves as an outgroup.

All nairovirus sequences obtained in the NS runs were identified as CCHFV, present in 20 (50%) of the pools (Table 1). Mapped reads or contig counts of 1–1,752 were noted, with 80.7–98.7% identities to closely related CCHFV sequences (Supplementary Table 3). We obtained near-complete coding region contigs of all CCHFV genome segments in one pool (p58–37) and L or S segment contigs in others (Supplementary Table 3). In the maximum likelihood phylogenetic analysis, the sequences clustered with CCHFV genogroup VI (Europe 2 or AP-92-like) isolates (Figure 2), which has recently been reclassified as the Aigai virus (AIGV) (Papa et al., 2022). Originally reported from Greece, AIGV was also reported from Albania, Bulgaria, Kosovo, and Turkey, and *R. bursa* ticks were suggested as the primary vector. Based on reports from Turkey and Iran, AIGV is a human pathogen, with the capacity to produce mild or severe CCHF-like disease (Midilli et al., 2009; Salehi-Vaziri et al., 2016).

**Figure 3:**
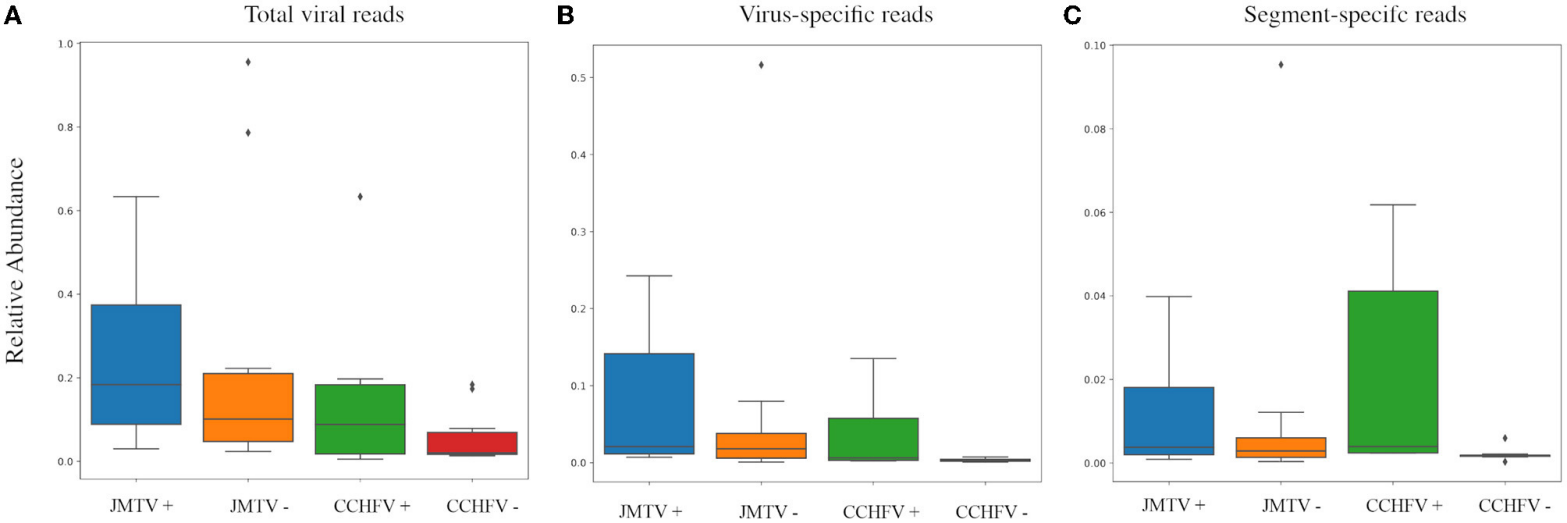
Comparison of the total virus, specific virus, and PCR-targeted segment abundances in samples with detectable JMTV and CCHFV in NS. Abundances are presented as a relative ratio to total reads **(A)** and virus reads **(B, C)**. Values in each group are given range + standard deviation, with outliers indicated with rhombuses. No statistically significant difference was observed between any PCR-positive and negative groups.

We further compared JMTV and CCHFV detection rates by PCR and NS (Table 2). Six pools (15%) were positive using the single round generic nairovirus PCR, whereas the nested JMTV PCR identified nine pools (22.5%) as positive (Supplementary Tables 2, 3). In NS, all PCR-positive pools produced contigs of every genome segment from each virus (Table 2). Detection by NS was more frequent for both viruses with a statistical significance of 95% confidence. However, no significant difference between total virus, specific virus, or targeted segment read numbers was observed in PCR-positive and negative pools with virus detection by NS (Figure 3).

**Table 2:** Crosstable of virus detection by PCR and nanopore sequencing.

**PCR**		**Nanopore sequencing**			
		**Crimean-Congo hemorrhagic fever virus**		**Jingmen tick virus**	
		**Positive**	**Negative**	**Positive**	**Negative**
Crimean-Congo hemorrhagic fever virus	Positive	6	0	4	2
	Negative	10	24	15	19
Jingmen tick virus	Positive	5	4	9	0
	Negative	11	20	10	21

### 3.3. Mivirus findings

*Mivirus* is a recently established genus within the *Chuviridae* family (order *Jingchuvirales*) (Di Paola et al., 2022). Initially described by NGS in arthropods (Li et al., 2020), jingchuviruses are documented to be broadly distributed in major arthropod subphyla, and further associated with eucestodes, fish, helminths, mammals, nematodes, reptiles, and sea anemones. They have highly diverse, non-segmented, segmented, and/or circular genomes, varying ORF configurations and preliminary evidence of genome integration and human infections (Di Paola et al., 2022). We detected two miviruses in tick pools. BTV3 stands out as the most frequently detected virus, present in 33 (82.5%) of the pools (Table 1). In NS, read counts ranging between 1 and 468 with 83.5–99.6% BLASTN identities to the BTV3 strain previously reported from Anatolia were observed (Ergünay et al., 2020). Complete virus replicase, glycoprotein, and nucleoprotein ORF contigs could be retrieved from certain pools (Supplementary Table 4). Phylogeny construction using partial replicase, glycoprotein, and nucleoprotein contig alignments showed a clustering of the Anatolian sequences, distinct from other BTV3 strains and miviruses (Supplementary Figure 1). In addition to BTV3, a single read with 89.2% identities to Xinjiang mivirus 1 replicase was detected in a pool of *R. sanguineus* ticks (Supplementary Table 4).

### 3.4. Phlebovirus findings

We detected 1–18 reads or contigs mapped to phleboviruses in 24 (60%) of the tick pools (Supplementary Table 5). The sequences comprised L and S segments of the virus genome, with BLASTN identities of 90.6–99.0 and 80.1–98.8% to various phleboviruses, respectively. Tick-associated phleboviruses identified in the pools include Brown dog tick phlebovirus 2 (BDTP2), phlebovirus Strandja, tick phlebovirus Anatolia, and Lesvos virus (Table 1). Strandja and Anatolia phleboviruses were mainly reported in *Rhipicephalus* ticks from Bulgaria and Turkey (Emanet et al., 2019; Ohlendorf et al., 2019), while BDTP2 appears widespread, detected in China, and Trinidad and Tobago as well as the Balkans (Sameroff et al., 2019; Bratuleanu et al., 2022; Guo et al., 2022). Lesvos virus was originally reported from *Hae. parva* ticks from the Greek island of Lesbos (Papa et al., 2016). Vertebrate infections have not previously been documented for any of these viruses. Phylogeny construction based on partial polymerase sequences on the L genome segment revealed the grouping of the sequences with related viruses from the Balkans and Turkey (Supplementary Figure 2).

### 3.5. Other viruses

Finally, we detected sequences of an *Orthomyxovirus* (Quaranjavirus), a *Peribunyavirus* (*Ixodes ricinus* bunyavirus-like 1 virus), and an unclassified *Riboviria* (Butler's Creek virus) in three tick pools (Table 1). In a pool of male *R. sanguineus* samples collected from infested canines, three reads identified as *Quaranjavirus* sp. were detected. The reads comprised 113, 171, and 244 base pairs and showed 92.37–98.2% identities to a novel Quaranjavirus PB1 sequence, recently reported in ticks from Romania (Bratuleanu et al., 2022). Quaranjaviruses are members of the *Orthomyxoviridae* family (Walker et al., 2019), and the initial isolates were described in cases of febrile diseases, with exposure in indigenous populations (Taylor et al., 1966). They have been historically documented in argasid ticks and their vertebrate hosts, primarily avian species. Some quaranjaviruses are associated with avian mortality and demonstrate pathogenicity under experimental conditions (Allison et al., 2015; Shearn-Bochsler et al., 2017; Mourya et al., 2019). Documentation of various quaranjaviruses in several hard tick species in the genera *Amblyomma, Haemaphysalis, Ixodes*, and *Rhipicephalus* including this study suggest that unrecognized tick-borne infections may be occurring (Cholleti et al., 2018; Sameroff et al., 2021; Bratuleanu et al., 2022; Kobayashi et al., 2022). Furthermore, two reads (279 and 767 base pairs) in a *Hae. parva* pool revealed 70–72.8% identity to the Butler's Creek virus, reported from pooled *Haemaphysalis bancrofti* nymphs from Australia. Finally, a single read with 70% identity to the M segment of *Ixodes ricinus* associated bunyavirus-like 1 virus reported from Croatia (Sameroff et al., 2022), was identified in a *R. bursa* pool.

## 4. Discussion

Our findings demonstrate that NS sequencing robustly detects tick-borne viruses in pooled ticks, with detection sensitivities significantly exceeding broad-range and nested PCR. Unlike targeted generic, specific, or multiplexed amplification, metagenome sequencing has the capability of detecting novel targets along with known pathogens without the need for prior information. However, during metagenome sequencing of field-collected arthropods, non-pathogenic microbial, host and environmental nucleic acids may mask viral sequences of interest, resulting in reduced pathogen detection sensitivity (Greninger et al., 2015; Kumar et al., 2017; Petersen et al., 2019). Sample pooling, a common practice in vector screening, is likely to increase background signal. To overcome this problem, various enrichment approaches including centrifugation/filtration, depletion of ribosomal RNA, targeted virus sequence capture, random priming, and non-specific amplification have been used (Brinkmann et al., 2016; Petersen et al., 2019). In this study, we employed a straightforward cDNA-based library preparation protocol, except for the DNase treatment of purified nucleic acids. This approach not only enabled the identification of the main targets JMTV and CCHFV, but recovered several other tick-associated viruses as well, with near-complete genome sequences obtained in samples with a sufficient number of mapped reads. Despite the relatively low number of total and virus reads due to our barcoding strategy, the longer read lengths generated by NS provided genome-wide virus sequence information, enabling reliable phylogeny construction in most of the detected viruses.

Due to the public health threat potential emergency and the lack of efficacious therapeutic or preventive measures, CCHFV is included among the pathogens targeted in the World Health Organization research and development blueprint (Mehand et al., 2018). It is widely distributed in Asia, Africa, and Southeast Europe, where the primary vector ticks of the *Hyalomma* genus are prevalent. CCHFV further exhibits a striking genome diversity, with several distinct genotypes based on viral genome segments and associated with geographic location, and with the possibility for recombination among strains (Lukashev et al., 2016). The recently reclassified AIGV was considered of low virulence, with few documented cases (Papa et al., 2022). This is further supported by the findings of a recent *in vitro* study, where diminished viral L protein expression was observed in AIGV (Pickin et al., 2022). The diagnostic or screening nucleic acid and serologic assays in current use are mostly unable to differentiate between these viruses; therefore, the impact of AIGV in CCHFV-associated disease incidence and epidemiology is hard to assess. Despite different primary hosts (*Hyalomma* vs. *Rhipicephalus*), tick species with detectable CCHFV or AIGV may overlap significantly in endemic regions, and genetic exchange resulting in altered virulence is also possible. Hence, adequate tools to diagnose and monitor AIGV are required, and our findings further indicate NS as a robust approach for this purpose.

Our NS-based metagenome approach further revealed a JMTV prevalence of 60% in the screened tick pools, and the subsequent phylogeny construction revealed clustering with those previously characterized in Anatolia and the Balkans. JMTV and related viruses (often referred to as Jingmenviruses) are included in the *Flaviviridae* family based on non-structural protein similarities, despite having segmented genomes (Simmonds et al., 2019; Temmam et al., 2019). Currently, known Jingmenvirus genomes comprise two large phylogenetic clades and are associated with tick-vertebrate or insect hosts (Colmant et al., 2022). Since its initial description in China, JMTV sequences have been detected in samples from many locations in Asia, Africa, Europe, and America (Temmam et al., 2019; Colmant et al., 2022). Reported with high prevalence in *Rhipicephalus microplus* ticks from various regions, JMTV has also been identified in many species of *Rhipicephalus, Amblyomma, Dermacentor, Haemaphysalis, Hyalomma*, and *Ixodes* ticks throughout the globe, but ecological data on transmission cycles are scant. In addition to ticks, virus RNA has been documented in bats, cattle, rodents, tortoises, individuals with febrile disease, and those co-infected with CCHFV (Emmerich et al., 2018; Jia et al., 2019; Temmam et al., 2019; Colmant et al., 2022). By antibody detection, JMTV exposure has been identified in cattle and humans from Asia and Europe. Despite its widespread presence in many regions, human JMTV infections are reported sporadically, presumably due to a common clinical presentation with well known tick-borne infections and lack of commercial diagnostic assays. Similarly, another Jingmenvirus named Alongshan virus, was detected in patients with tick-associated febrile diseases with subsequent seroconversion and evidence for exposure in domestic animals and humans (Temmam et al., 2019; Wang et al., 2019; Colmant et al., 2022). These findings demonstrate that at least two Jingmen viruses qualify as tick-borne emerging viruses and considering their ubiquitous distribution further confirmed in this study; they must be investigated in individuals with febrile diseases of unknown etiology associated with tick bites.

The broad-range detection provided by the metagenomic approach also enabled the detection of additional viruses, including two miviruses (BTV3 and Xinjiang mivirus 1), four phleboviruses (BDTP2, Anatolia, Strandja, and Lesvos phleboviruses), and other RNA viruses (Table 1). Interestingly, BTV3 infections were observed at an unprecedented frequency of 82.5%. BTV3 and tick phleboviruses appear to be a ubiquitous virus in Anatolia, having been detected in various tick species (Dinçer et al., 2017; Brinkmann et al., 2018; Emanet et al., 2019; Ergünay et al., 2020). Tick phleboviruses are also widely distributed in Asia, Europe, and America (Ohlendorf et al., 2019; Sameroff et al., 2019; Bratuleanu et al., 2022; Guo et al., 2022). As vertebrate *in vivo* experiments failed to propagate these viruses, they are currently considered non-virulent residents of the tick virome (Emanet et al., 2019). Other RNA viruses identified in the study have also been reported in tick metagenome investigations (Pettersson et al., 2017; Sameroff et al., 2022). Despite the lack of evidence for human or veterinary health impact of these viruses, indirect effects on the replication of the pathogenic viruses in vector ticks and potential genetic exchange among strains should be considered. NS further facilitated the first documentation of Quaranjaviruses in Anatolia, Turkey—a significant finding that warrants further biosurveillance due to their potential as animal and human pathogens.

Several shortcomings of the current study should be addressed. First, information on the number of infected individuals in each pool was lacking; therefore, assessments on individual ticks or mixed infections could not be performed. We did not use a CCHFV quantitative-PCR approach, as it may not ensure accurate detection of virus genotypes including AIGV in circulation and multiple genotypes. A more informative comparison would have been possible by parallel sequencing of spiked pools with a known copy number of viral genomes. During NS, we did not carry out a real-time data interpretation for virus detection, but an end-point analysis of the sequencing data instead. NS was reported to be capable of generating 12% of the Ross River virus reads in a single infected mosquito after 10 min and 87.3% in 10 h (Batovska et al., 2017). Follow-up studies are underway to calculate timelines for reliable pathogen detection and genotyping on individual and pooled ticks. A limitation of the NS-based metagenomics for vector-borne viruses is the lack of established controls, optimized for all potential target pathogens. Moreover, assessments of detection thresholds and sensitivity, as well as the correlation of read counts with standard quantitative nucleic acid detection assays are required. Operational costs and expertise required for assay performance and data interpretation should also be considered. However, metagenome-based surveillance provided a much broader view of the viruses in circulation, as exemplified by tick pools in this study. As affordable, field-friendly sequencing platforms become widespread, many of the current limitations are likely to be circumvented.

In conclusion, this proof-of-concept study revealed that metagenomic NS with standard approaches could surpass the sensitivity of PCR amplification in tick-borne viruses and generate genome-wide data for reliable phylogeny construction. The utility of NS could be particularly useful for monitoring pathogens in tick vectors or human/animal clinical samples in hot-spot regions for zoonotic spillover.

## Data availability statement

The datasets presented in this study can be found in online repositories. The names of the repository/repositories and accession number(s) can be found below: https://www.ncbi.nlm.nih.gov/genbank/, SRR21504630-SRR21504591; https://www.ncbi.nlm.nih.gov/genbank/OP432043–OP432053.

## Author contributions

KE, ED, and Y-ML: conceptualization. KE and ED: data curation. KE, ED, SAJ, BPB, SPN, MOT, BO, IS, and OFG: formal analysis. NLA, JPG, and Y-ML: funding acquisition and resources. ED, SPN, MOT, BO, IS, OFG, and DDR-W: investigation. H-ML and LJ: methodology. Y-ML: project administration and supervision. KE: writing—original draft. KE and Y-ML: writing—review and editing. All authors contributed to the article and approved the submitted version.

## References

[B1] Allison A. B. Ballard J. R. Tesh R. B. Brown J. D. Ruder M. G. Keel M. K. (2015). Cyclic avian mass mortality in the northeastern United States is associated with a novel orthomyxovirus. J. Virol. 89, 1389–1403. 10.1128/JVI.02019-14 25392223 PMC4300652

[B2] Altschul S. F. Gish W. Miller W. Myers E. W. Lipman D. J. (1990). Basic local alignment search tool. J. Mol. Biol. 215, 403–410. 10.1016/S0022-2836(05)80360-2 2231712

[B3] Apanaskevich D. A. Horak I. G. (2008). The genus *Hyalomma* Koch, 1844: V. Reevaluation of the taxonomic rank of taxa comprising the *H. (Euhyalomma)* marginatum Koch complex of species (Acari: Ixodidae) with redescription of all parasitic stages and notes on biology. Int. J. Acarol. 34, 13–42. 10.1080/01647950808683704

[B4] Batovska J. Lynch S. E. Rodoni B. C. Sawbridge T. I. Cogan N. O. (2017). Metagenomic arbovirus detection using MinION nanopore sequencing. J. Virol. Methods. 249, 79–84. 10.1016/j.jviromet.2017.08.019 28855093

[B5] Bratuleanu B. E. Temmam S. Munier S. Chretien D. Bigot T. van der Werf S. (2022). Detection of phenuiviridae, chuviridae members, and a novel quaranjavirus in hard ticks from Danube Delta. Front. Vet. Sci. 9, 863814. 10.3389/fvets.2022.863814 35498749 PMC9044029

[B6] Brinkmann A. Dinçer E. Polat C. Hekimoglu O. Hacioglu S. Földes K. (2018). A metagenomic survey identifies Tamdy orthonairovirus as well as divergent phlebo-, rhabdo-, chu- and flavi-like viruses in Anatolia, Turkey. Ticks. Tick. Borne Dis. 9, 1173–1183. 10.1016/j.ttbdis.2018.04.017 29728337

[B7] Brinkmann A. Nitsche A. Kohl C. (2016). Viral metagenomics on bloodfeeding arthropods as a tool for human disease surveillance. Int. J. Mol. Sci. 17, 1743. 10.3390/ijms17101743 27775568 PMC5085771

[B8] Buchfink B. Reuter K. Drost H. G. (2021). Sensitive protein alignments at tree-of-life scale using DIAMOND. Nat. Methods. 18, 366–368. 10.1038/s41592-021-01101-x 33828273 PMC8026399

[B9] Cholleti H. Hayer J. Mulandane F. C. Falk K. Fafetine J. Berg M. (2018). Viral metagenomics reveals the presence of highly divergent quaranjavirus in *Rhipicephalus* ticks from Mozambique. Infect. Ecol. Epidemiol. 8, 1478585. 10.1080/20008686.2018.1478585 29868166 PMC5974704

[B10] Colmant A. M. G. Charrel R. N. Coutard B. (2022). Jingmenviruses: Ubiquitous, understudied, segmented flavi-like viruses. Front. Microbiol. 13, 997058. 10.3389/fmicb.2022.997058 36299728 PMC9589506

[B11] Danecek P. Bonfield J. K. Liddle J. Marshall J. Ohan V. Pollard M. O. (2021). Twelve years of SAMtools and BCFtools. Gigascience. 10, giab008. 10.1093/gigascience/giab008 33590861 PMC7931819

[B12] De Coster W. D'Hert S. Schultz D. T. Cruts M. Van Broeckhoven C. (2018). NanoPack: visualizing and processing long-read sequencing data. Bioinformatics. 34, 2666–2669. 10.1093/bioinformatics/bty149 29547981 PMC6061794

[B13] Di Paola N. Dheilly N. M. Junglen S. Paraskevopoulou S. Postler T. S. Shi M. (2022). Jingchuvirales: a New Taxonomical Framework for a Rapidly Expanding Order of Unusual Monjiviricete Viruses Broadly Distributed among Arthropod Subphyla. Appl Environ Microbiol. 88:e0195421. 10.1128/aem.01954-21 35108077 PMC8939347

[B14] Dinçer E. Brinkmann A. Hekimoglu O. Hacioglu S. Földes K. Karapinar Z. (2017). Generic amplification and next generation sequencing reveal Crimean-Congo hemorrhagic fever virus AP92-like strain and distinct tick phleboviruses in Anatolia, Turkey. Parasit Vectors. 10, 335. 10.1186/s13071-017-2279-1 28705183 PMC5513282

[B15] Dinçer E. Timurkan M. Ö. Oguz B. Sahindokuyucu I. Sahan A. Ekinci M. (2022). Several Tick-Borne Pathogenic Viruses in Circulation in Anatolia, Turkey. Vector Borne Zoonotic Dis. 22:148–158. 10.1089/vbz.2021.0082 35133905

[B16] Dulanto Chiang A. Dekker J. P. (2020). From the pipeline to the bedside: advances and challenges in clinical metagenomics. J. Infect Dis. 221, 331–340. 10.1093/infdis/jiz151 31538184 PMC7325616

[B17] Emanet N. Kar S. Dinçer E. Brinkmann A. Hacioglu S. Farzani T. A. (2019). Novel tick phlebovirus genotypes lacking evidence for vertebrate infections in Anatolia and Thrace, Turkey. Viruses. 11, 703. 10.3390/v11080703 31374842 PMC6723390

[B18] Emmerich P. Jakupi X. von Possel R. Berisha L. Halili B. Günther S. (2018). Viral metagenomics, genetic and evolutionary characteristics of Crimean-Congo hemorrhagic fever orthonairovirus in humans, Kosovo. Infect. Genet. Evol. 65, 6–11. 10.1016/j.meegid.2018.07.010 30006045

[B19] Ergünay K. Dinçer E. Kar S. Emanet N. Yalçinkaya D. Polat Dinçer P. F.. (2020). Multiple orthonairoviruses including Crimean-Congo hemorrhagic fever virus, Tamdy virus and the novel Meram virus in Anatolia. Ticks Tick. Borne. Dis. 11, 101448. 10.1016/j.ttbdis.2020.101448 32723637

[B20] Estrada-Pena A. Bouattour A. Walker C. (2004). Ticks of Domestic Animals in the Mediterranean Region. 1st ed. Zaragoza: University of Zaragoza Press.

[B21] Filippova N. A. (1997). Fauna of Russia and Neighbouring Countries, Arachnoidea, Volume IV, Issue 5, Ixodid Ticks of Subfamily Amblyomminae. St. Petersburg: Nauka Publishing House.

[B22] Greninger A. L. Naccache S. N. Federman S. Yu G. Mbala P. Bres V. (2015). Rapid metagenomic identification of viral pathogens in clinical samples by real-time nanopore sequencing analysis. Genome Med. 7, 99. 10.1186/s13073-015-0220-9 26416663 PMC4587849

[B23] Guo J. J. Lin X. D. Chen Y. M. Hao Z. Y. Wang Z. X. Yu Z. M. (2020). Diversity and circulation of Jingmen tick virus in ticks and mammals. Virus Evol. 6:veaa051. 10.1093/ve/veaa051 33976906 PMC8097133

[B24] Guo L. Ma J. Lin J. Chen M. Liu W. Zha J. (2022). Virome of *Rhipicephalus* ticks by metagenomic analysis in Guangdong, southern China. Front. Microbiol. 13, 966735. 10.3389/fmicb.2022.966735 36033874 PMC9403862

[B25] Honig J. E. Osborne J. C. Nichol S. T. (2004). The high genetic variation of viruses of the genus *Nairovirus* reflects the diversity of their predominant tick hosts. Virology. 318, 10–16. 10.1016/j.virol.2003.09.021 14972529

[B26] Huson D. H. Beier S. Flade I. Gorska A. El-Hadidi M. Mitra S. (2016). MEGAN community edition—interactive exploration and analysis of large-scale microbiome sequencing data. PLoS Comput. Biol. 12, e1004957. 10.1371/journal.pcbi.1004957 27327495 PMC4915700

[B27] Jia N. Liu H. B. Ni X. B. Bell-Sakyi L. Zheng Y. C. Song J. L. (2019). Emergence of human infection with Jingmen tick virus in China: a retrospective study. EbioMedicine. 43, 317–24. 10.1016/j.ebiom.2019.04.004 31003930 PMC6557783

[B28] Kobayashi D. Kuwata R. Kimura T. Faizah A. N. Higa Y. Hayashi T. (2022). Detection of Quaranjavirus-Like Sequences from *Haemaphysalis hystricis* ticks collected in Japan. Jpn. J. Infect. Dis. 75, 195–198. 10.7883/yoken.JJID.2021.129 34470960

[B29] Kumar A. Murthy S. Kapoor A. (2017). Evolution of selective-sequencing approaches for virus discovery and virome analysis. Virus Res. 239, 172–179. 10.1016/j.virusres.2017.06.005 28583442 PMC5819613

[B30] Kumar S. Stecher G. Li M. Knyaz C. Tamura K. (2018). MEGA X: molecular evolutionary genetics analysis across computing platforms. Mol. Biol. Evol. 35, 1547–1549. 10.1093/molbev/msy096 29722887 PMC5967553

[B31] Li C. X. Shi M. Tian J. H. Lin X. D. Kang Y. J. Chen L. J. (2020). Unprecedented genomic diversity of RNA viruses in arthropods reveals the ancestry of negative-sense RNA viruses. Elife. 4, e05378. 10.7554/eLife.05378 25633976 PMC4384744

[B32] Li H. (2018). Minimap2: pairwise alignment for nucleotide sequences. Bioinformatics. 34, 3094–3100. 10.1093/bioinformatics/bty191 29750242 PMC6137996

[B33] Lukashev A. N. Klimentov A. S. Smirnova S. E. Dzagurova T. K. Drexler J. F. Gmyl A. P. (2016). Phylogeography of crimean congo hemorrhagic fever virus. PLoS One. 11, e0166744. 10.1371/journal.pone.0166744 27880794 PMC5120814

[B34] Mehand M. S. Al-Shorbaji F. Millett P. Murgue B. (2018). The WHO RandD Blueprint: 2018 review of emerging infectious diseases requiring urgent research and development efforts. Antiviral Res. 159, 63–67. 10.1016/j.antiviral.2018.09.009 30261226 PMC7113760

[B35] Midilli K. Gargili A. Ergonul O. Elevli M. (2009). The first clinical case due to AP92 like strain of Crimean-Congo Hemorrhagic Fever virus and a field survey. BMC Infect. Dis. 9, 90. 10.1186/1471-2334-9-90 19515251 PMC2700115

[B36] Mourya D. T. Yadav P. D. Nyayanit D. A. Majumdar T. D. Jain S. Sarkale P. (2019). Characterization of a strain of quaranfil virus isolated from soft ticks in India. Is quaranfil virus an unrecognized cause of disease in human and animals. Heliyon. 5, e01368. 10.1016/j.heliyon.2019.e01368 30957047 PMC6431747

[B37] Ohlendorf V. Marklewitz M. Kopp A. Yordanov S. Drosten C. Junglen S. (2019). Huge diversity of phleboviruses in ticks from Strandja Nature Park, Bulgaria. Ticks. Tick. Borne. Dis. 10, 697–703. 10.1016/j.ttbdis.2019.03.001 30871930

[B38] Papa A. Kontana A. Tsioka K. Saratsis A. Sotiraki S. (2016). Novel phlebovirus detected in *Haemaphysalis parva* ticks in a Greek island. Ticks. Tick. Borne. Dis. 8, 157–160. 10.1016/j.ttbdis.2016.10.012 27818182

[B39] Papa A. Marklewitz M. Paraskevopoulou S. Garrison A. R. Alkhovsky S. V. Avšič-Županc T. (2022). History and classification of Aigai virus (formerly Crimean-Congo haemorrhagic fever virus genotype VI). J. Gen. Virol. 103, 4. 10.1099/jgv.0.001734 35412967 PMC10026732

[B40] Petersen L. M. Martin I. W. Moschetti W. E. Kershaw C. M. Tsongalis G. J. (2019). Third-generation sequencing in the clinical laboratory: exploring the advantages and challenges of nanopore sequencing. J. Clin. Microbiol. 58, e01315–e01319. 10.1128/JCM.01315-19 31619531 PMC6935936

[B41] Pettersson J. H. Shi M. Bohlin J. Eldholm V. Brynildsrud O. B. Paulsen K. M. (2017). Characterizing the virome of *Ixodes ricinus* ticks from northern Europe. Sci. Rep. 7, 10870. 10.1038/s41598-017-11439-y 28883464 PMC5589870

[B42] Pickin M. J. Devignot S. Weber F. Groschup M. H. (2022). Comparison of crimean-congo hemorrhagic fever virus and aigai virus in life cycle modeling systems reveals a difference in l protein activity. J. Virol. 96, e0059922. 10.1128/jvi.00599-22 35695578 PMC9278617

[B43] Quer J. Colomer-Castell S. Campos C. Andrés C. Piñana M. Cortese M. F. (2022). Next-generation sequencing for confronting virus pandemics. Viruses. 14, 600. 10.3390/v14030600 35337007 PMC8950049

[B44] Quick J. Loman N. J. Duraffour S. Simpson J. T. Severi E. Cowley L. (2016). Real-time, portable genome sequencing for Ebola surveillance. Nature. 530, 228–232. 10.1038/nature16996 26840485 PMC4817224

[B45] Russell J. A. Campos B. Stone J. Blosser E. M. Burkett-Cadena N. Jacobs J. L. (2018). Unbiased strain-typing of arbovirus directly from mosquitoes using nanopore sequencing: a field-forward biosurveillance protocol. Sci. Rep. 8, 5417. 10.1038/s41598-018-23641-7 29615665 PMC5883038

[B46] Salehi-Vaziri M. Baniasadi V. Jalali T. Mirghiasi S. M. Azad-Manjiri S.. (2016). The first fatal case of Crimean-Congo hemorrhagic fever caused by the AP92-like strain of the Crimean-Congo hemorrhagic fever virus. Jpn. J. Infect. Dis. 69, 344–346. 10.7883/yoken.JJID.2015.533 26902209

[B47] Sameroff S. Tokarz R. Charles R. A. Jain K. Oleynik A. Che X. (2019). Viral diversity of tick species parasitizing cattle and dogs in trinidad and tobago. Sci Rep. 9, 10421. 10.1038/s41598-019-46914-1 31320705 PMC6639388

[B48] Sameroff S. Tokarz R. Jain K. Oleynik A. Carrington C. V. F. Lipkin W. I. (2021). Novel quaranjavirus and other viral sequences identified from ticks parasitizing hunted wildlife in Trinidad and Tobago. Ticks. Tick. Borne Dis. 12, 101730. 10.1016/j.ttbdis.2021.101730 33957484

[B49] Sameroff S. Tokarz R. Vucelja M. Jain K. Oleynik A. Boljfeti,ć M. (2022). Virome of *Ixodes ricinus, Dermacentor reticulatus*, and *Haemaphysalis concinna* ticks from Croatia. Viruses. 14, 929. 10.3390/v14050929 35632671 PMC9146755

[B50] Shearn-Bochsler V. Ip H. S. Ballmann A. Hall J. S. Allison A. B. Ballard J. (2017). Experimental Infection of Common Eider Ducklings with Wellfleet Bay Virus, a Newly Characterized Orthomyxovirus. Emerg Infect Dis. 23:1958–1965. 10.3201/eid2312.160366 28841405 PMC5708229

[B51] Simmonds P. Becher B. Bukh J. Gould E. A. Meyers G. Monath T. (2019). ICTV virus taxonomy profile: flaviviridae. J. Gen. Virol. 98, 2–3. 10.1099/jgv.0.000672 28218572 PMC5370391

[B52] Taylor R. M. Hurlbut H. S. Work T. H. Kingston J. R. Hoogstraal H. (1966). Arboviruses isolated from *Argas* ticks in Egypt: quaranfil, chenuda, and nyamanini. Am. J. Trop. Med. Hyg. 15, 76–86. 10.4269/ajtmh.1966.15.76 5901633

[B53] Temmam S. Bigot T. Chrétien D. Gondard M. Pérot P. Pommelet V. (2019). Insights into the Host Range, Genetic Diversity, and Geographical Distribution of Jingmenviruses. Ecol. Evol. Sci. 4, e00645–e00619. 10.1128/mSphere.00645-19 31694898 PMC6835211

[B54] Trinh P. Zaneveld J. R. Safranek S. Rabinowitz P. M. (2018). One health relationships between human, animal, and environmental microbiomes: a mini-review. Front. Public Health. 6, 235. 10.3389/fpubh.2018.00235 30214898 PMC6125393

[B55] Walker A. R. Bouattour A. Camicas J. L. Estrada-Pena A. (2003). Ticks of Domestic Animals in Africa: A Guide to Identification of Species, 1st ed. Edinburgh: Bioscience Reports.

[B56] Walker B. J. Kerians J. E. Horak I. G. (2000). The Genus Rhipicephalus (Acari, Ixodidae): A Guide to the Brown Ticks of the World, revised ed. Cambridge: Cambridge University Press.

[B57] Walker P. J. Siddell S. G. Lefkowitz E. J. Mushegian A. R. Adriaenssens E. M. Alfenas-Zerbini P. (2019). Changes to virus taxonomy and the international code of virus classification and nomenclature ratified by the international committee on taxonomy of viruses. Arch. Virol. 164, 2417–2429. 10.1007/s00705-019-04306-w 31187277

[B58] Wang Z. D. Wang B. Wei F. Han S. Z. Zhang L. Yang Z. T. (2019). A new segmented virus associated with human febrile illness in China. N. Engl. J. Med. 380, 2116–2125. 10.1056/NEJMoa1805068 31141633

[B59] Wick R. R. Judd L. M. Gorrie C. L. Holt K. E. (2017). Completing bacterial genome assemblies with multiplex MinION sequencing. Microb. Genom. 3, e000132. 10.1099/mgen.0.000132 29177090 PMC5695209

[B60] Wick R. R. Judd L. M. Holt K. E. (2019). Performance of neural network basecalling tools for Oxford Nanopore sequencing. Genome. Biol. 20, 129. 10.1186/s13059-019-1727-y 31234903 PMC6591954

[B61] Yu Z. M. Chen J. T. Qin J. Guo J. J. Li K. Xu Q. Y. (2020). Identification and characterization of Jingmen tick virus in rodents from Xinjiang, China. Infect Genet Evol. 84:104411. 10.1016/j.meegid.2020.104411 32531517 PMC7283072

