# Supplementary figures and images for: Impact of nanopore-based metagenome sequencing on tick-borne virus detection
Source: Front Microbiol. 2023 May 30;14:1177651. doi: 10.3389/fmicb.2023.1177651 (PMC10267750; doi:10.3389/fmicb.2023.1177651)

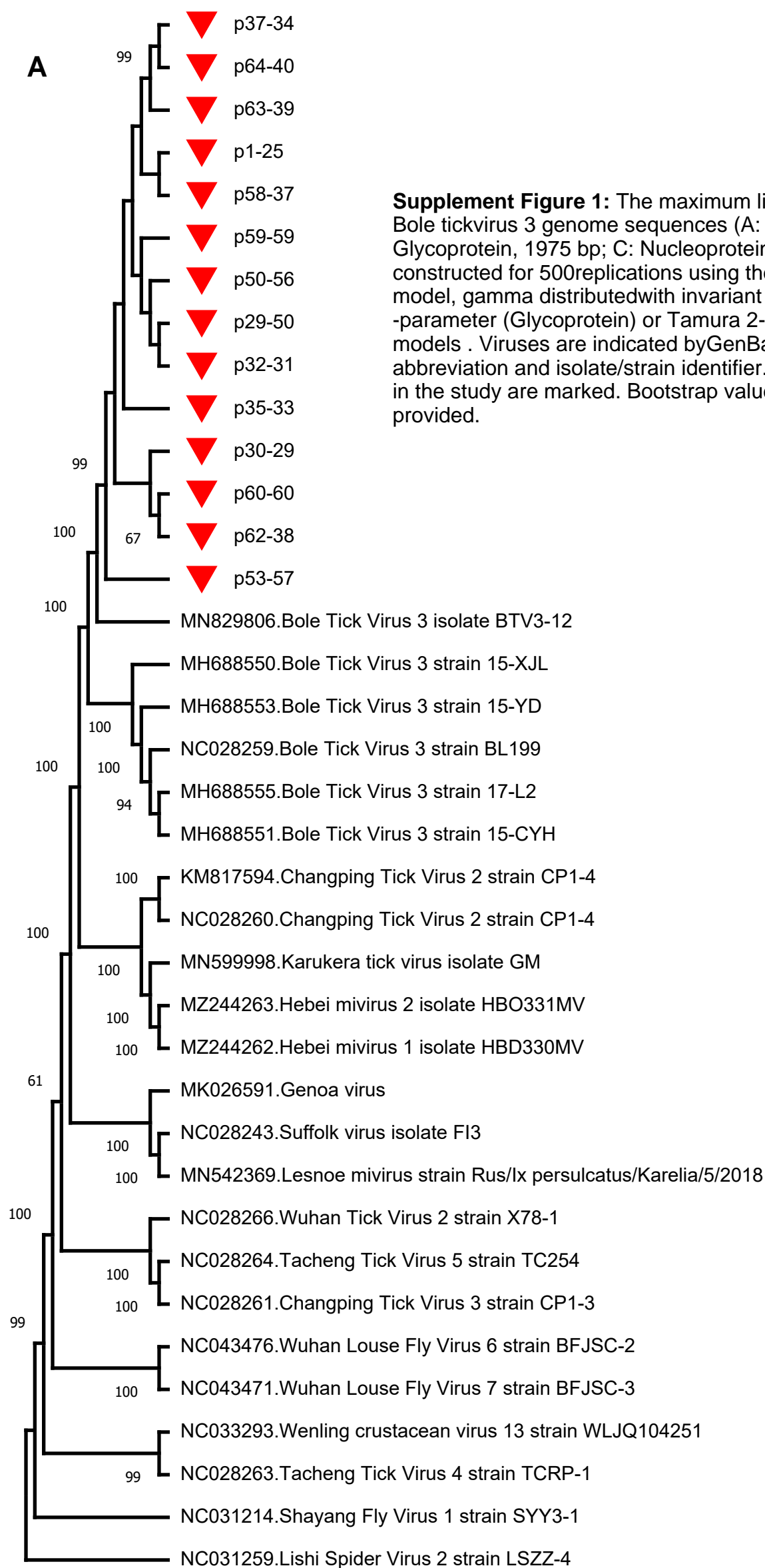

**B**

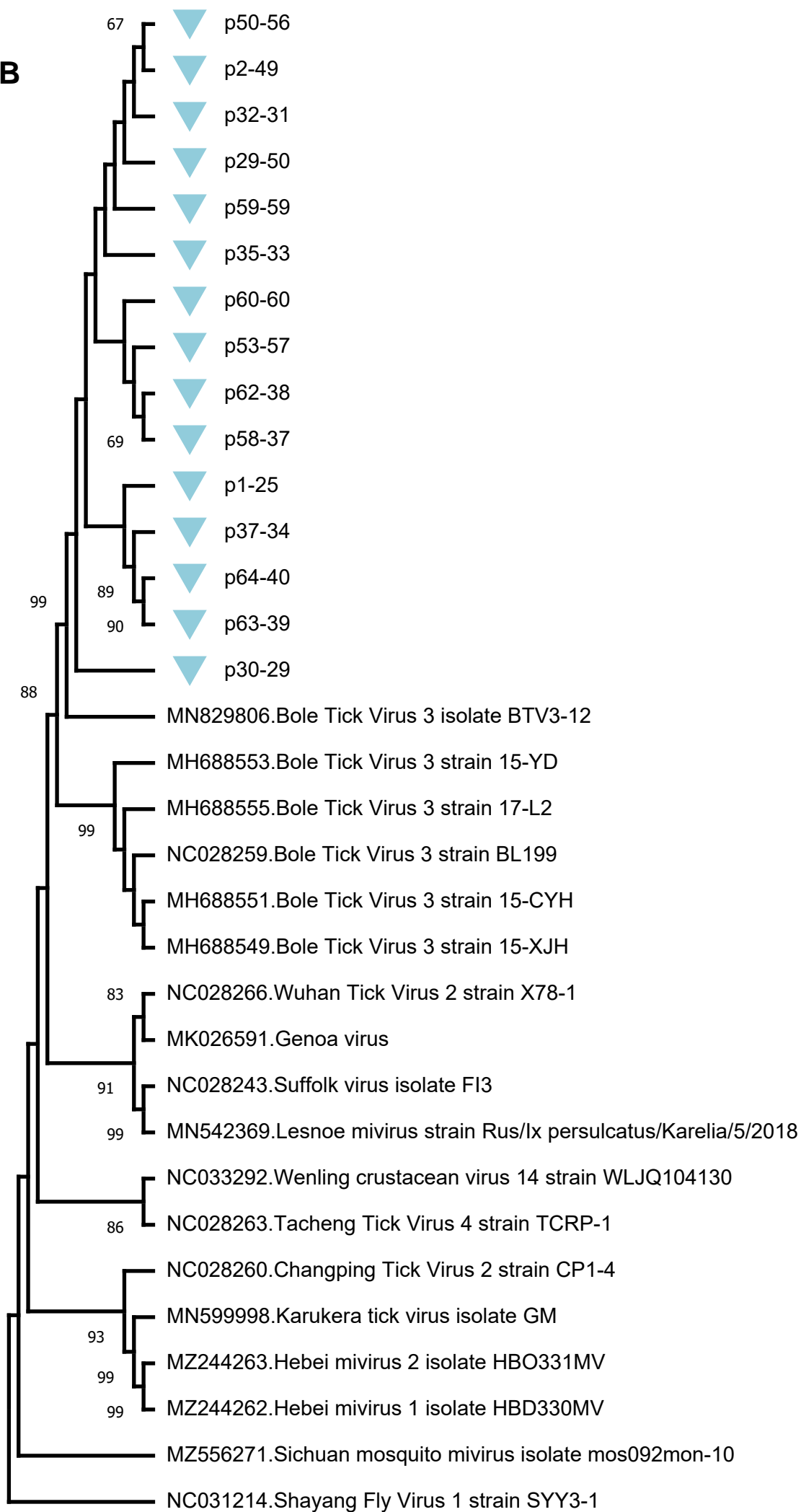

C

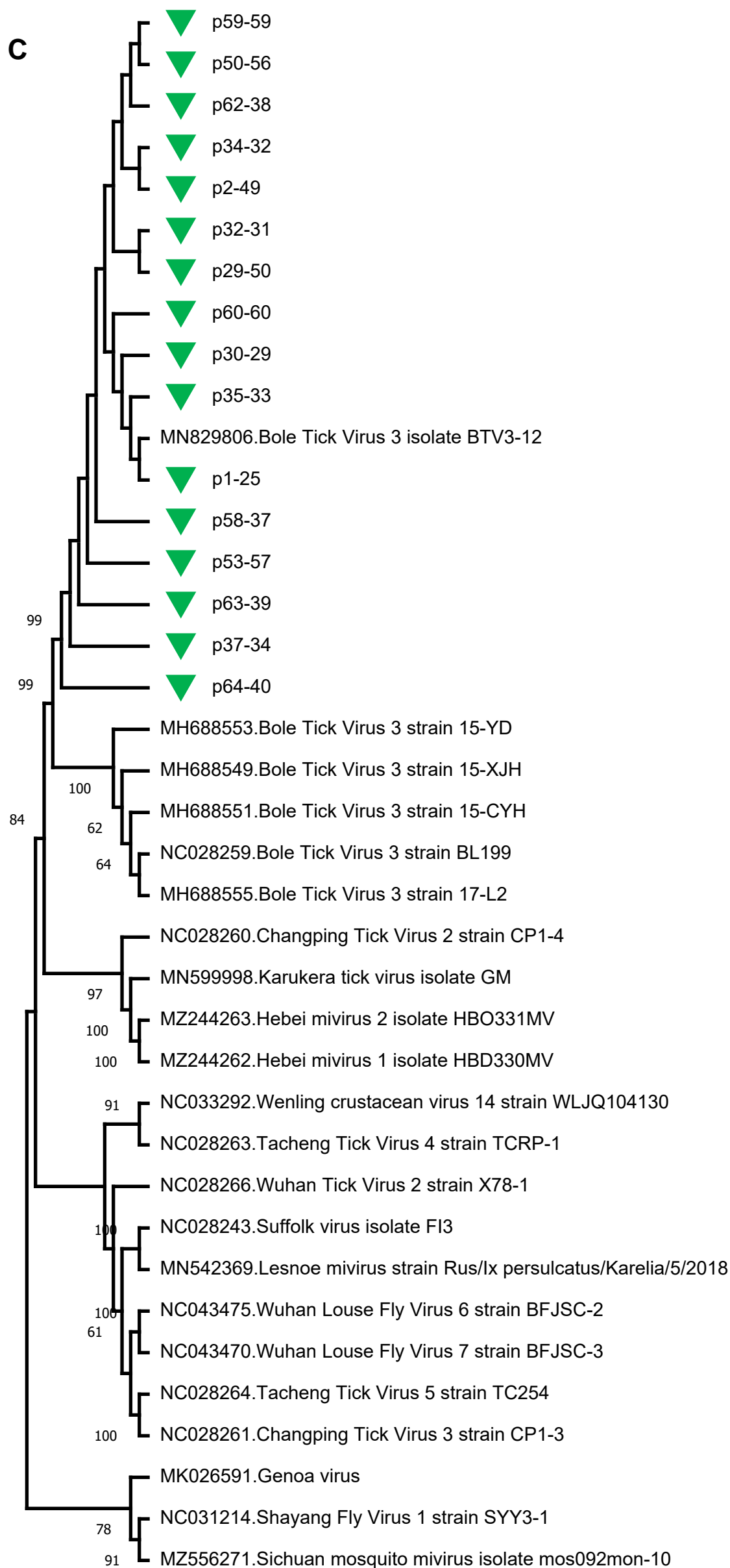

Supplement: Supplementary file 1 [file Data_Sheet_1.PDF]
