# Supplementary material for: Impact of nanopore-based metagenome sequencing on tick-borne virus detection
Source: Front Microbiol. 2023 May 30;14:1177651. doi: 10.3389/fmicb.2023.1177651 (PMC10267750; doi:10.3389/fmicb.2023.1177651)
Supplement: Supplementary file 2 [file Data_Sheet_2.PDF]

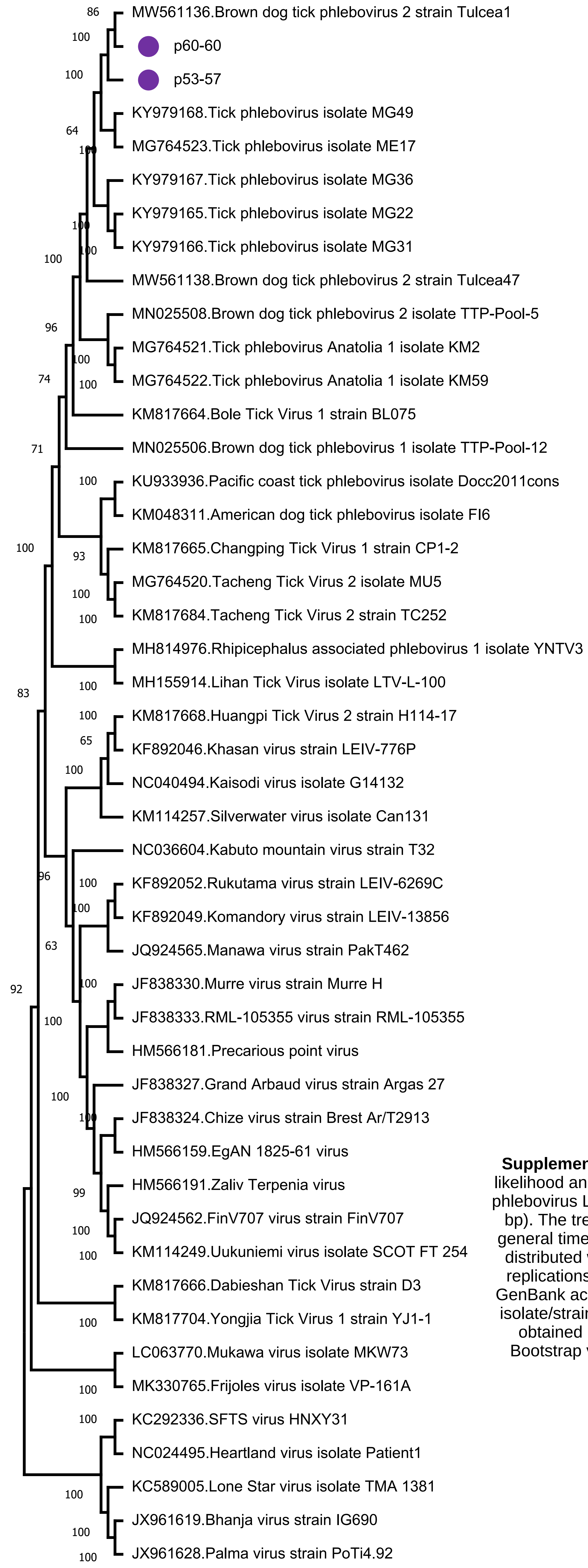

Supplement Figure 2: The maximum likelihood analysis of the tick-associated phlebovirus L segment sequences (1016 bp). The tree is constructed using the general time reversable model, gamma distributed with invariant sites for 500 replications. Viruses are indicated by GenBank accession number, name and isolate/strain identifier. The sequences obtained in the study are marked. Bootstrap values higher than 60 are provided.
